# Missing from the data, missing from the design: feminist co-design for equitable hormonal contraception symptom tracking

**DOI:** 10.3389/fgwh.2026.1864517

**Published:** 2026-08-10

**Authors:** Rachael Hughson-Gill, Chloe Curtis, Lisa Iversen, Catie Varley, Abigail Needham, Rebecca L. Mawson

**Affiliations:** 1 College of Health and Science, Lincoln Institute for Rural and Coastal Health, University of Lincoln, Lincoln, United Kingdom; 2 School of Anthropology & Museum Ethnography, Institute of Social & Cultural Anthropology, University of Oxford, Oxford, United Kingdom; 3 Academic Primary Care, Institute of Applied Health Sciences, University of Aberdeen, Aberdeen, United Kingdom; 4 Centre for Regional Economic and Social Research, Sheffield Hallam University, Sheffield, United Kingdom; 5 NIHR HealthTech Research Centre (HRCs), Sheffield Teaching Hospital, Devices for Dignity, The NIHR HRC in Long Term Conditions, Sheffield, United Kingdom; 6 Primary Care Research Group, School of Medicine and Population Health, University of Sheffield, Sheffield, United Kingdom

**Keywords:** app development, co-design, data ethics, equalities, femtech, hormonal contraception, intersectionality, underserved populations

## Abstract

**Introduction:**

Hormonal contraception is used by millions globally, yet side effects remain poorly understood and underrepresented in clinical research and digital health tools. The femtech sector has largely failed to serve marginalised communities, including ethnic minority groups, neurodivergent individuals, and those with lived experience of mental health challenges. This study explored these diverse perspectives through the co-design of T.H.E (The Hormone Effect) app, a digital tool for collecting data on hormonal contraception side effects, to ensure it serves these populations and enables the generation of more diverse and representative datasets.

**Methods:**

Five co-design workshops were conducted in South Yorkshire, UK (May–June 2025), engaging 24 participants across five community groups: a Muslim women's group, a Muslim mums’ group, a neurodiversity support group, women with lived experience of mental health challenges, and university students. Participants reviewed a low-fidelity prototype of T.H.E app alongside existing cycle-tracking apps, engaging in facilitated discussion and annotation. Data was analysed using deductive qualitative content analysis.

**Results:**

Five themes emerged. Participants called for flexible, inclusive, and trauma-informed data collection, raising concerns about fixed identity categories, clinical language, and the potential harms of existing mental health questionnaires. Trust and transparency were central, with significant concern about data commercialisation and privacy, particularly regarding abortion and pregnancy loss. Language and visual design were identified as key barriers and enablers of engagement. Participants also advocated strongly for reciprocal value through meaningful data visualisation and culturally relevant health information.

**Discussion:**

Women's health data collection is an embodied, emotionally experienced, and culturally situated practice. Moving beyond extractive approaches toward transparent, trauma-informed, and user-centred design is essential for equitable digital health technology. Future work should prioritise iterative development of T.H.E app and its integration into clinical pathways to address persistent data gaps in hormonal contraception research.

## 1 Introduction

Hormonal contraception is used by millions of individuals globally, yet the side effects associated with its use remain poorly understood and significantly under-researched (1–3). Even though women commonly use hormonal contraception for around three quarters of their reproductive lives, research into the individual experience of side effects remains scarce, and those that do exist reveal how varied, complex, and deeply personal these experiences can be (1). Mental health impacts, bleeding disturbances, and headaches are among the most reported concerns, yet many users feel dismissed or unheard by healthcare practitioners when raising them (1, 4). This persistent gap between lived experience and clinical acknowledgement reflects a broader pattern in which women's health has been systematically deprioritised, underfunded, and under-researched. Specifically, no existing digital health tool has been designed to systematically capture patient-reported hormonal contraception side-effect data in a way that generates an equitable, research-usable evidence base; this is the specific data gap that the present study addresses.

The inequities in women's healthcare are not experienced uniformly. Marginalised communities (including those from ethnic minority backgrounds, neurodivergent individuals, and those with lived experience of mental health challenges) are chronically underrepresented in both clinical research and the datasets that inform it (5–8). Drawing on Crenshaw's foundational framework, intersectionality is used here to understand how overlapping systems of oppression (including gender, race, disability, and socioeconomic position) compound health inequities and shape whose experiences are rendered visible or invisible in research (9). This pattern reflects what Mitchell et al. describe as the ‘inverse research law': the tendency for those who face the greatest burden of ill health to be the least represented in the evidence base that is meant to serve them (7, 10). When biases are embedded at multiple stages of the research process, from agenda-setting to recruitment to data interpretation, they become entrenched in clinical practice, perpetuating rather than addressing health disparities. This is not incidental; it reflects longstanding structural exclusions in how medical knowledge is produced and whose bodies and experiences are considered worthy of study (5, 11). Feminist scholars have argued that medical categories and prior research frameworks can fail to capture women's embodied realities, particularly when they diverge from dominant, Western, cisgender norms, thereby further entrenching data gaps for minoritised groups (4, 5). Digital health technology has the potential to address these concerns and close this data gap at scale by considering side effects in new ways (8, 12).

The emergence of femtech has seen a growing sector of digital health technologies increasingly oriented toward women's reproductive health, menstrual tracking, and contraception management. However, critics have argued that the femtech industry is driven primarily by the logic of financial capital rather than health equity, with investment concentrated in scalable, profitable applications that serve higher-income users while marginalising the health needs of those already underserved (5). No apps currently exist specifically designed to track hormonal contraception side effects. Existing cycle tracking tools, such as Flo™, Clue™, and Apple™'s built-in health feature, were not designed to track hormonal contraception side effects or to generate equitable datasets. In addition, user-facing functions are often behind paywalls and data is inaccessible to researchers, rendering these tools unsuitable for research addressing health inequity. Without deliberate attention to equity in design, technology-enabled data collection risks reproducing the same exclusions present in traditional research.

This study addresses that gap through the co-design and development of T.H.E (The Hormone Effect) app, a digital research tool designed to generate equitable, representative data on hormonal contraception side effects. A feminist co-design approach was chosen specifically to meet this gap: it is understood here as both a methodological practice and an ethical commitment. The project aimed to actively and meaningfully involves marginalised communities who would use or be impacted by an end product, recognising that they bring expert lived and embodied knowledge that cannot be replicated from the outside (13).

This feminist co-design approach is particularly important in the context of hormonal contraception, where the body of evidence itself reflects whose experiences have historically been centred and whose have been overlooked. Community-based recruitment methods were employed to engage groups traditionally underrepresented in research, including Muslim women, neurodivergent individuals, and students - a purposive sample designed to foreground intersectional diversity. Sheffield, England's fourth largest city with a diverse population, provided a meaningful context in which to explore how design decisions land differently across communities with distinct cultural, social, and experiential perspectives (14).

This paper presents findings from those co-design workshops, examining community perspectives on the app's design and their broader implications for equitable, ethical, and accessible technology-enabled data collection in women's health. This work builds on a growing body of research exploring contraceptive access, experience, and data inequity for underrepresented populations - including work that has sought to actively challenge the power imbalances embedded in how research with and about marginalised communities is designed and conducted (7, 10). In doing so, it contributes to emerging calls for digital health tools that move beyond extractive data practices toward genuinely reciprocal relationships with users - particularly those whose experiences have historically been rendered invisible in both clinical research and the femtech landscape (15).

The methods below detail how a feminist co-design approach was applied to translate this aim into a structured, community-led workshop process.

## 2 Materials and method

### 2.1 Feminist co-design methodology

This study is underpinned by a feminist co-design methodology. Co-design is defined as a collective practice of design that actively and meaningfully involves representatives of communities who would use or be impacted by the end-product, in this case T.H.E app (13). This active community involvement is associated with community-based participatory research (16) and this study can be considered a design-specific instance. Co-design can disrupt power structures (17), and here it is combined with feminist approach that focuses on equity and justice. This involves working with communities historically marginalised, underrepresented, ignored, and ill-treated within research and digital technology. Our use of this methodology centres the voices of women and transmen from marginalised communities within design, recognising their expert lived and embodied knowledge of experiences (17, 18). This knowledge has a depth, nuance, and intimacy that is difficult to fully understand by those who do not experience it. In this instance, co-design enables experiences to be shared, and ideas to be generated, debated, and developed from an intimate understanding of how they could sit within people's lives and affect their experiences. Positionality and ways to bring differing experiential knowledge are important to consider when designing for diversity of experience outside that of personal socio-cultural experience and the complex socio-cultural landscape of hormonal contraception and femtech.

These commitments directly shaped the methodological choices described below; recruitment prioritised existing community relationships over convenience sampling, workshops were held in community-nominated venues rather than clinical or university settings. Our facilitation used open annotation and discussion rather than structured questionnaires, so that participants’ own framings, not predefined categories, drove the analysis.

### 2.2 Participant recruitment

Community-based recruitment was used for a purposive and opportunistic sample of women and transmen, primarily from marginalised communities. Men and trans women were excluded from this study due focus on female specific hormonal contraception. No age, or fertility, or sexuality restriction were applied within sampling diversity of experience was welcomed. Recruitment was conducted in collaboration with community groups and members (19), supported by established and ongoing relationships in the Sheffield and Rotherham areas. This opportunistic approach drew on existing trust within these communities and enabled engagement with populations that are traditionally underrepresented in research (16, 19, 20). An intersectional range of identities was represented in the sample through engagement with the following community groups: a Muslim women's group, a Muslim mums’ group, a neurodiversity support group, women with lived experience of mental health challenges, and university students. All participants provided informed consent to be part of the research.

### 2.3 T.H.E app prototype development

**Figure 1:**
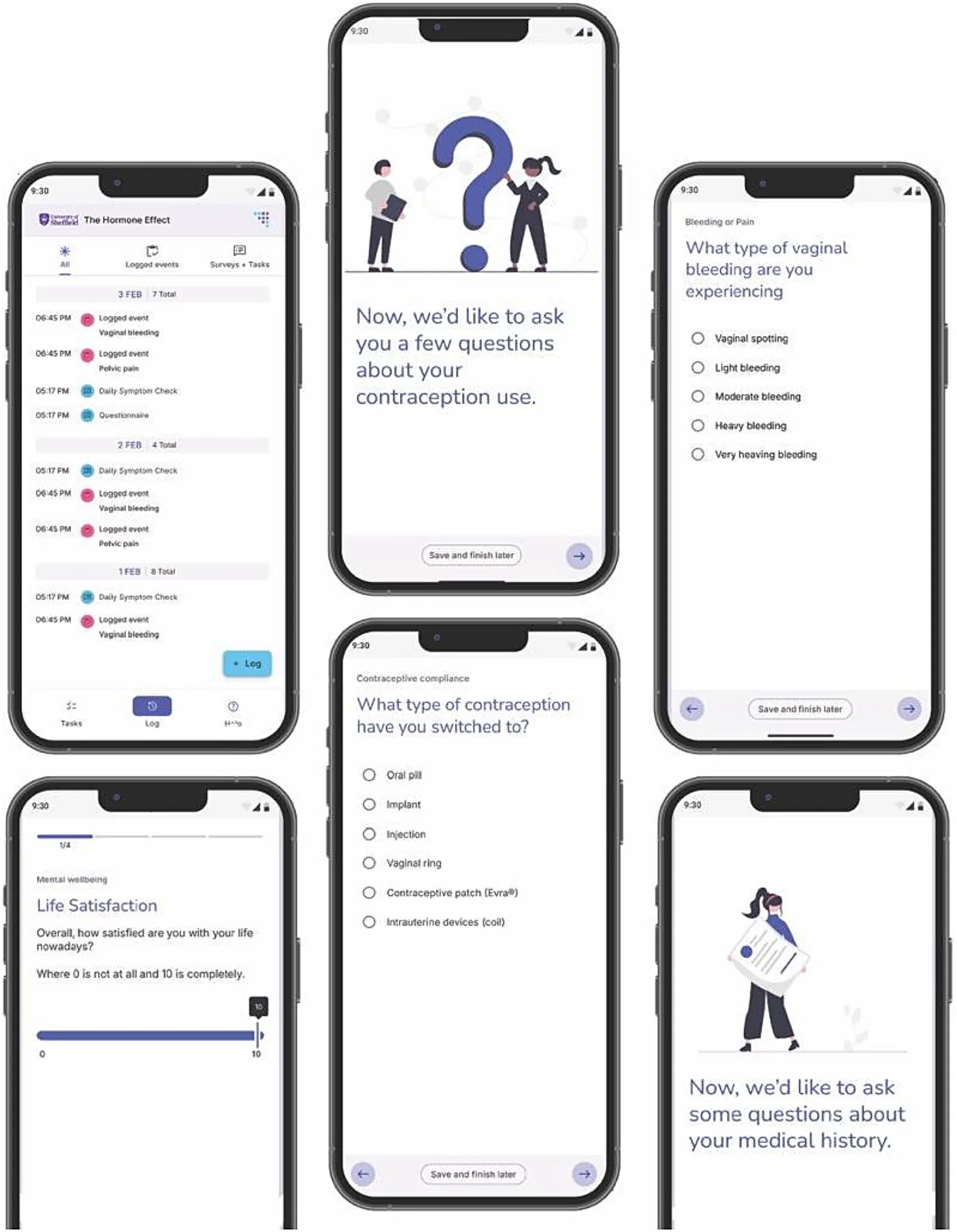
Visual excerpt of T.H.E app prototype.

An initial low-fidelity version of T.H.E app, (Figure 1) was developed to be used within the co-design workshops. A low-fidelity prototype is an early-stage representation of an interface that sits purposefully in between concept and finished product, allowing for meaningful directed community input (8). These prototypes take a step into the ‘real’, making concepts tangible and easier to understand. They are also far enough away from a finished product, with allocated time and funds for user-research, that participants’ feedback and ideas can support meaningful improvement.

The initial T.H.E app Alpha prototype was designed and developed by RM, the project's Principal Investigator (**PI**), and DAISER, digital medical platform developers (https://www.daiser.com/). This was based on RM's pre-existing research and clinical experience. T.H.E app prototype requirements (such as data to be collected) were informed by clinical practice regarding contraception provision, the medical research experience of the PI, input from community engagement, and the project's existing research programme with input from community engagement and building on our current research program (21, 22).

The basic prototype had four main sections: 1- login and onboarding, 2 - personal and medical information, 3 - landing page, 4 - symptom recording. Login and onboarding are the first interactions to set up the app, in which the user will log in and be provided with starter information such as app navigation and permissions. Personal and medical information collects data about the user demographics, as well as contraception use, medical condition(s), and pregnancy history. The landing page is the user interface that presents upcoming and past data collection, and a help section for using the app. Finally, the symptom recording will ask users to provide data on vaginal bleeding (menstrual, spotting, and other), pain (pelvic and headaches), digestion (nausea, constipation) and mental well-being [using the Office for National Statistics (ONS 4) personal wellbeing questions] (23). Symptom recording aims to collect data on how symptoms and side effects may develop and change when using hormonal contraception. The ONS 4 was recommended by the South Yorkshire Digital Health Hub academic lead for all apps within the cohort.

### 2.4 Co-design workshop procedure

#### 2.4.1 Study setting

Co-design workshops were 2 h long and took place between 8th May and 12th June 2025, and were conducted in South Yorkshire, UK. Sheffield is England's fourth largest city, located in the North of England, with a diverse population home to people identifying as white (79.1%), Asian or Asian British (9.6%), Black (4.6%) and mixed or other ethnic groups (24). The workshops took place in spaces known and used by the communities involved (Table 1). Running workshops in community spaces followed recommended practice to remove practical and psychological barriers to participation, support psychological safety, and address power hierarchies through researchers being invited guests (25). Researchers sought to further promote a setting of psychological safety for new and existing relationships through intentionally relaxed and personable interactions and environment.

**Table 1:** Participant involvement and sample representation.

Group	Number of participants	Ethnicities	Neurodivergence	Age range	Workshop location
1 - Muslim women's group	5	Asian/Asian British, Black African	Not recorded	18–58	Mosque
2 - Muslim mums’ group	6	Asian/British Asian, British Arab.	Not recorded	21–35	Café
3 - Neurodiversity support group	6	White	ADHD, autism or ADHD and autism	18–55	Library (where support group is held)
4 - Women with lived experience of mental health challenges	4	White other, white, mixed, Tamil	Not recorded	22–27	Design studio
5 - Students	3	Not recorded	Not recorded	19–23	University study space

#### 2.4.2 Priming activity: existing apps

Participants took part in a priming activity, an approach used to immerse participants in the subject matter, build confidence and to prepare for the next activity (26). No hormonal contraception side effects specific tracking apps exist so participants were given printed static screenshots of existing menstruation, and hormonal cycle tracking apps: Flo™, Clue™, and Apple™'s iPhone in-built health feature. Although more general symptom-tracking apps (e.g., Bearable and Medisafe) are available, the selected apps were chosen due to their specific focus on women's health and the likelihood that participants would be familiar with them, thereby supporting building confidence with critical engagement as a priming activity. Participants were asked to explore them, annotating the printouts, and engaging in group discussion on their views, preferences, and opinions on their design and how they would or would not be usable and acceptable. This priming activity aimed to give participants a comparative context to explore T.H.E app while also fostering a collaborative and creative mindset and working dynamic.

#### 2.4.3 Prototype feedback and ideas for improvement: T.H.E app

Participants then moved to the main component of the workshop, giving community input on the design of T.H.E low-fidelity prototype. Participants were given paper-based images of T.H.E app prototype, as foreshadowed in the priming activity, and asked to explore them. Digital prototypes were shown on iPads, but due to functionality issues, it was difficult to engage with them. Participants were invited to annotate the printed screenshots (writing, drawing, and using stickers) and engage in group conversations about their views, preferences, and opinions on the design, and on whether it would or would not be usable and acceptable. In addition, participants were also invited to give ideas and suggestions on how they think T.H.E app could be improved and adapted, drawing from learnings in the previous activity, lived experiences, and their own creativity and problem solving (27, 28). Conversation, enquiry, and idea generation were prompted, where needed and appropriate, by semi-structured open-ended questions based on key design principles.

### 2.5 Data collection

Data collection was primarily participant led, where they recorded feedback and ideas onto the app printouts. The facilitators (RHG/RM) also took supplementary notes to capture discussion and observations. In addition, where appropriate and when additional verbal consent was obtained, group discussions were audio-recorded to support analysis but were not transcribed verbatim.

### 2.6 Data analysis

Analysis was done (RHG/RM) using deductive qualitative content analysis with a directed approach (29). An initial coding framework was predefined based on the four digital infrastructure sections of the app (1- login and onboarding, 2 - personal and medical information, 3 - landing page, 4 - symptom recording); key cross-cutting principles of interaction design (readability and understandability, interface architecture and visual hierarchy, visual language); and further design suggestions that did not fit into pre-existing categories. Analysis was interpretive rather than quantitative; code frequencies were not calculated, as the objective was thematic understanding that enabled user feedback and ideas to directly inform app design and development.

The units of analysis consisted of words, phrases, or drawings that represented distinct feedback or idea concepts. Each unit was systematically documented, including the group number, data source (annotation, audio recording, facilitator notes), time stamp for audio data, and a summarised interpretation of the feedback or idea concept. These units were then deductively coded to the predefined framework. Following initial coding, the data was revisited for a second-order thematic abstraction. This involved identifying higher-order themes that captured feedback and ideas cutting across multiple framework categories.

Analytic rigour was enhanced through peer debriefing and reflexive practice. Peer debriefing between RHG and RM was conducted to ensure coded data was reflective of participants’ feedback and ideas. Reflexivity was maintained throughout the analytical process, exploring diverging viewpoints in the data and critically considering the coders’ perspectives (30). By reflecting on positionality, and ensuring the results remained reflective of the diverse participant group, the analysis aimed to minimise research bias. This practice of seeking to address bias is essential within this application of co-design that aims to understand the needs, desires, and design for diverse, marginalised, and underserved groups of women and transmen.

### 2.7 Ethical approval

This study received ethical approval from the University of Sheffield ethics committee (date 08/04/2025 and reference number 067198). Specific effort was given to ensure ethical procedure and practice within this project, approaching with sensitivity and awareness of power and responsibility. Care was taken throughout participant engagement due to the nature of the project, exploring what can be potentially sensitive or difficult subject matter, and working with marginalised groups.

## 3 Results

This section first outlines the participant sample before presenting the themes that emerged from analysis, it builds on the feminist co-design methodology and analytic approach described above.

There were five co-design workshops involving a total of 24 participants, 23 women and one transman. Demographics are summarised in Table 1; not all participants provided demographic data. Recruitment achieved diversity in neurological, ethnic, and age representation.

Data analysis resulted in identification of five higher-order themes that capture these diverse groups’ feedback and ideas on the use of T.H.E app to track hormonal contraception side-effects.

### 3.1 Providing sensitivity, inclusivity, and flexibility in data collection

Participants highlighted the need for data collection with the app to be flexible, inclusive, and sensitive. It was found that fixed demographic categories may not reflect the lived realities of identity, including multiple ethnic backgrounds and non-binary gender identities. Identity questions were felt to be both affirming and uncomfortable; while one participant valued the consideration of ethnicity, others noted, as seen below, that individuals from ethnic minority backgrounds may not be comfortable disclosing this information.

“Difficult with the race questions… I’d be more willing, but I know like for example my mum would probably still say no because it's like why do you need to know that.” – Discussion quote from student group

Discussions also showed a desire for flexibility in data input associated with contraception use and hormonal side effect symptoms. One group suggested adding an open box to add their own reasons for using or changing contraception outside the listed options. Within the group discussion, it was suggested that additional symptoms and the ability to add their own should be possible. They also suggested that they would like to have the ability to add more details about symptoms and factors that may affect them, such as types and locations of pain, missed contraception, and an open note function.

“I also put I think there should be like an option for you know women to add their own notes… I think like with symptoms and stuff it just says like abdominal cramps and stuff, but it doesn't say specifically what that woman is going through.”- Discussion quote from Muslim women's group

Participants highlighted several current questions that were perceived as inappropriate and potentially harmful. Questions relating to pregnancy history, including number of pregnancies, miscarriages, still births and mode of birth, were discussed as potentially being difficult for women who had experiences of pregnancy loss. One participant also raised concerns about the cultural sensitivity of asking about abortions and caesarean birth, noting the stigmatising connotations within their culture.

“They really need to word these way better because like from, people from ethnic backgrounds who I’ve seen at least these questions are very sensitive, because it also comes with societal shame. Because if you have an abortion you are deemed as a failure back home, the same goes with c-sections.” - Discussion quote from student group

**Figure 2:**
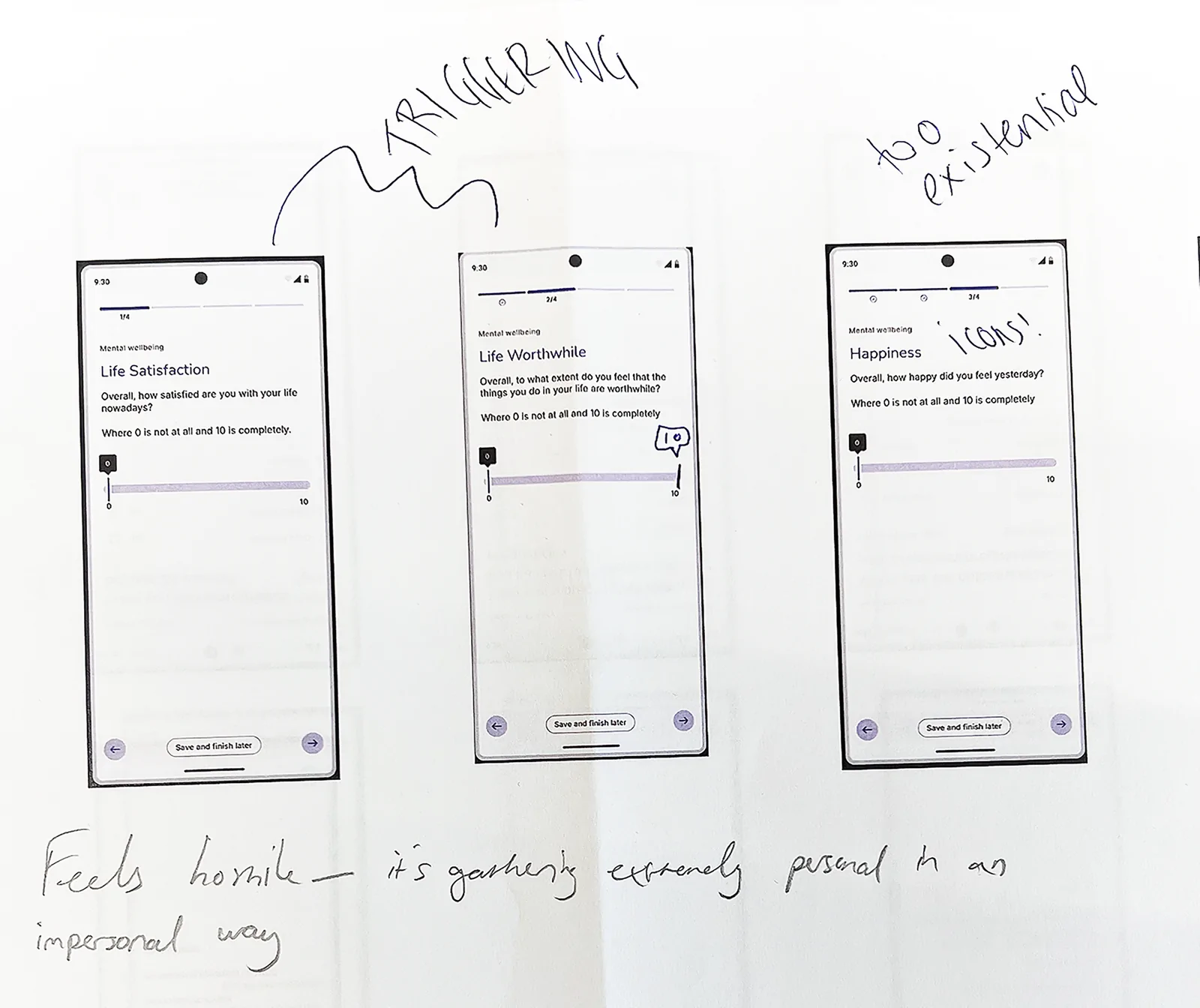
Example participant feedback on ONS well-being survey questionnaire.

Across all groups, participants emphasised the inappropriate nature of the use of ONS 4 well-being questions within the app, which were described as highly clinical and “triggering” (Figure 2) with the potential to negatively affect mental health and well-being. One group extended this issue questioning an app's capacity to appropriately respond to the disclosure of information of acute mental health distress. Participants suggested the need for trigger warnings, inclusion of support information, and emphasised the importance of being able to opt in or out of questions while using the app.

“TRIGGERING… too existential…Feels horrible – its gathering extremely personal [data] in an impersonal way” - Annotation excerpt from lived experience of mental health challenges group.

### 3.2 Fostering trust through transparency, data security, and privacy

**Figure 3:**
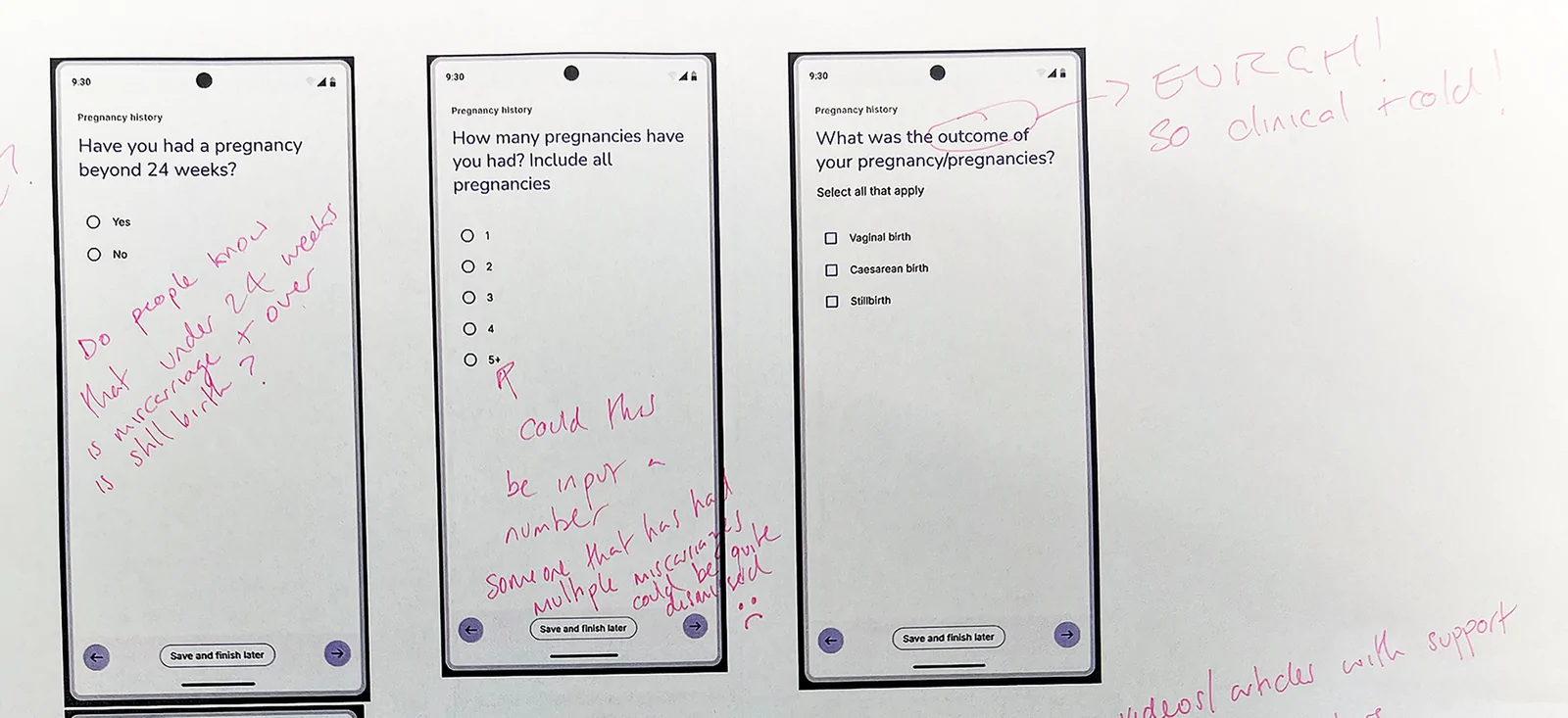
Example of challenges with language, readability and understandability.

Discussions throughout the workshops emphasised the need for transparency in data collection and management, as well as in the associated app permissions. When information was given in the prototype, such as the reasoning for push notification permissions, it was valued by participants. As shown in Figure 3, participants emphasised the need for clear information on why data was being collected, its relevance to the research, and the broader context of the research goals and background.

“I would feel more comfortable sharing this information if I knew why the app wanted it.”- Annotation excerpt from neurodiversity support group.

Also highlighted was the need for data privacy and clear information about how data is handled. Although participants’ views were mixed, with a small minority being comfortable with ‘cookies’ and the commercialisation of data, many were highly opposed to it. Discussion revealed a broader mistrust of femtech due to concerns about companies’ values and whether primary intentions are to support women or pursue commercial gain. Participants discussed unease about providing their data, specifically highlighting concerns relating to pregnancy loss and abortion, with many sharing fears related to current and potential changes to abortion rights.

“Are there assurances that info wouldn’t go to police/government lots of worries around police involvement about abortion laws” – Annotation excerpt from neurodiversity support group

The need for local data privacy, so as not to disclose information to people around them whilst using the app, was also discussed. They also conversed about the ability to edit any push notifications to disguise reminders associated with menstruation or hormonal contraception. Participants discussed the possibility of the app and content being ‘locked’, however, it was noted that this should be balanced so as not to further perceptions of women's health as taboo.

### 3.3 The use of language for readability and understandability

Discussions throughout the workshops identified language within the app content that may be difficult to understand. The need for clearer information on all medical terminology was noted, highlighting barriers to understanding caused by stigma, lack of public discussion and awareness around women's health, and the additional challenges for users whose first language is not English. Contraception methods and brand names were seen as a particular challenge, and it was suggested that a drop-down menu be included with medical and common names to ease use. It was found that some medical terms also have negative connotations. Discussion identified that ‘contraceptive compliance’ could imply surveillance, and ‘vaginal bleeding’ was ambiguous (as seen below when described as “terrifying”), with participants unclear whether it referred to menstruation, abnormal bleeding, or injury.

“We were told to type at an 8th grade level because that's like the standard of level of English that most people have, including immigrants or people that don't really speak English as their native language. So vaginal bleeding could sound terrifying to someone that didn't speak English.” – Discussion quote from student group

Discussion further emphasised challenges with medical and research reasoning behind questions. Within pregnancy history questions, the relevance of 24 weeks as a defining difference between miscarriage and stillbirth may not be known. Confusion around self-definition across questions also emerged, with participants unclear how bleeding should be categorised, and if mental health conditions needed to be current and or diagnosed. Similar uncertainty arose regarding if neurodivergence needed to be diagnosed or self-defined. Participants also highlighted that they found the timings within the ONS 4 questionnaire unclear and inconsistent, referring to: nowadays, overall, and yesterday. This led to confusion; participants in both the neurodivergent and lived-experience-of-mental-health-challenges groups highlighted that it can be difficult to recall emotional experiences. Multiple groups also highlighted a desire for question framing and language use that was warm and friendly in tone rather than overly medical. An example of a group highlighting multiple challenges with language can be seen in Figure 3.

“Do people know that under 24 weeks is miscarriage+over is still birth?.. could this be input a number someone that has had multiple miscarriages could be quite dismissed ☹… EURCH! So clinical +cold!”- Annotation excerpt from Neurodiversity support group

### 3.4 Creating appeal and communicating trust through visual identity and branding

The importance of trust in the app was emphasised throughout the conversations, which highlighted the role of visual identity and branding. It was suggested that the onboarding, the first interaction with the app, should have clear T.H.E branding, give more information about the research, and be welcoming. One group emphasised that this welcome should present the research team behind the app and project.

“If I saw women's faces and a bit of background about them I would trust it more than this is very faceless… rather than we have some questions, like who are we, I want to see faces give me real human faces.”- Discussion quote from Neurodiversity support group

All groups engaged in in-depth discussions about the tone and visual language of the app, and conversations highlighted the need for balance to communicate trust while also being visually appealing. Across all groups, there was consistent feedback on the desire for more visuals within the app, such as the use of icons, images, and a mascot. There was also a shared emphasis on ensuring that imagery is relevant and presents diversity. Furthermore, the colour scheme and branding were negatively described as clinical and commercial. All groups emphasised the need for greater use of colour within the app design. While some suggested pastel tones, pink, and purple, other groups highlighted the importance of incorporating gender-neutral colour schemes with one group specifically stressing that this was essential for ensuring the app is inclusive of transmen. While there was a call for more visuals, conversely, the discussion also showed a desire for research-focused visual language. It was noted, as an example seen below, that this clearly signals that the app is part of a research project, whereas, by contrast, commercial-style designs were associated with concerns about data privacy and potential distrust.

“In a way I don't hate that it feels like a survey.. this is for a research study and you are giving someone else your data I kind of, in a way I kind of like that it feels like that… I mean in my opinion if it felt too much like Clue or like Flo it would feel like it was trying to make you forget that that's what it's for.” – Discussion quote from student group

### 3.5 Providing value for women using the app through data presentation, visualisation, and learning

Discussion within the groups demonstrated that participants felt the app should provide direct value to the user. All groups commented that their data should be presented back to them. Many groups valued the current time stamps on symptom logging, discussing that it would support learning about one's own body. This was particularly emphasised within the neurodivergent group and they indicated that it would support challenges with perception of time. There were detailed conversations around the benefit of data visualisation to support engagement and to identify trends and patterns within symptoms. Participants also suggested the potential for medical interpretation of the data, informing individuals if symptoms and bleeding are abnormal and if so, when to see a doctor. Within group discussion, participants expressed a desire for the app to support trend identification and provide symptom predictions, noting that hormonal symptoms can significantly impact day-to-day life.

“Your mood fluctuates more, really, really badly.. I would love to see that.. I don't need it to be spot on, but if you can predict for me how I’m going to feel when that ovulation hits that's going to feel great you know what I mean because at least then I can go maybe I need to be a bit careful with myself today.”- Discussion quote from neurodiversity support group

Participants suggested that the app could support learning about women's health, seen to be important particularly concerning aspects of women's health that are taboo, insufficiently taught, and rarely discussed. Several groups illustrated the perceived value in credible medical information, articles from lived experience, and a community space to facilitate peer learning and support. They also noted that content should be relevant to hormonal contraception and menstruation, with the written material and visuals informative and appropriate. In addition, the Muslim women's groups highlighted the importance of culturally specific content such as how spotting can affect prayers. It was also raised that these functions should remain accessible and not sit behind a paywall.

“It will tell you, which I really like about it as well, you have an abnormal cycle this month… then it will be like would you like to see more insights on this or learn more about this and I’ll be like okay and you’ll click into it and then they want a subscription so I’m like forget it.”- Discussion quote from Muslim women’s group

## 4 Discussion

This study explored minority groups’ interpretations, opinions, and ideas for improvement of T.H.E app prototype design to collect data on hormonal contraception side effects. Our finding show that data collection about experiences of hormonal contraception is embodied, emotionally experienced, and culturally situated, and that ethical, trauma-informed data collection practice is therefore a precondition for equitable femtech design, not an optional refinement. The sections below consider what this means for inclusive digital health design and where it departs from prevailing femtech norms.

### 4.1 Data collection as an embodied, emotional, and culturally situated practice

A central finding was that participants experience data collection as an embodied and emotional practice that is deeply culturally situated. This raises concerns about the appropriateness of highly medicalised and clinical framing in this context, as these approaches may be experienced **as** detached from and insufficiently responsive to lived realities of embodied data. A considerable finding is the potential harm caused by using the ONS 4 mental health and well-being questionnaire, which is often considered ‘*good for research’* due to its validation, wide use, and large existing dataset that provides a reference for comparison. However, these co-design results show that “*good for research*” is not always the same as “*good for the user”*. This has prompted change in language throughout T.H.E and change within emotional tracking language from ONS 4 to exploring alternative measures of mood/affect such as ecological momentary assessment (EMA) (31). This also contributes to wider ongoing discussions about the need for new forms of practice and understanding that encompass the experiential and medical (32). This tension has implications that extend well beyond this study. Where validated instruments are routinely embedded in digital health tools without consideration of their experiential impact, the tools themselves can become a site of harm.

The embodied and lived quality of bodily data, and the diversity of experiences this encompasses, has further implications in the design of digital data collection systems. This research highlights how fixed, pre-defined data categories can constrain participants’ responses and fail to capture lived realities. Data on hormonal side effects is not well recorded, especially those experienced by minority groups (1–3). As a result, when predefined data categorisation is based on existing knowledge risks overlooking lived experience, intersectional complexity, and further entrenching existing gaps and inequalities in the data (4).

### 4.2 Trust, transparency, and the political economy of women's health data

This research contributes to ongoing emphasis on the importance of data security and ethical governance in relation to women's health data. Increasing data commercialisation is eroding trust with forms of technology-enabled data collection and ethical questions are being raised about who owns women's data, who makes financial gain, and what it is used for (33, 34). The embodied aspects of women's health data also suggests that it is connected to wider politics about reproductive bodies. Participants raised particular concern about data related to abortion and pregnancy loss, which must be understood within the broader political context of (shifting) legality, for example, post Roe v. Wade, as well as cases in which femtech data has been used in legal proceedings against women (34). For many marginalised communities, distrust may be further shaped by the historic context of research, healthcare, technology, and legal justice systems, which have not only failed to serve but exploited and harmed minoritised communities (5, 10, 11).

The results highlight the need for ethical data collection, governance, and use, and underscore the importance of transparency of data practices, which is a focus in T.H.E app's future design iterations. This design iteration represents a shift away from viewing those using digital data collection systems as passive data sources, towards recognising them as people who can and should be able to make informed choices about the use of their data. This also reflects an ethical stance of non-extractive data practices that values the decisions of women as knowledge producers, that we believe should be reflected throughout research and design practice.

### 4.3 Readability and clarity as a prerequisite for equity in access and diversity of data

The use of language can present barriers to access and meaningful use of data collection systems. The findings illustrate that language and its use are multifaceted, carrying connotations as well as cultural and political meaning beyond their technical function. Results prompted change in language within T.H.E app's future design iterations (for example changing “what type of vaginal bleeding are you experiencing” to “how was your bleeding today?”), the use of text-to-speech, and integration of clear additional information for necessary medical language. These results also raise wider questions about medical language appropriateness and acceptability in the context of personal and sensitive embodied data (35).

The results further illustrated that clarity extends beyond simply understanding the words to also encompass understanding the underlying medical and research reasoning embedded within data collection. These results together support multidimensional conceptualisations of health literacy shaped by both comprehension, understanding what is being asked, and lived experience that shape how these are interpreted (3, 33).

This understanding of language and literacy is particularly important when designing for diversity and inclusion whereby language and lived realities may differ (36). The multi-layered nature of health literacy therefore presents challenges for equitable engagement which, if specific attention is not given to usability, risks further exacerbating existing inequity by reinforcing gaps in data and understanding related to contraception and women's health more widely.

### 4.4 Visual design as a mechanism for trust building and acceptability

**Figure 4:**
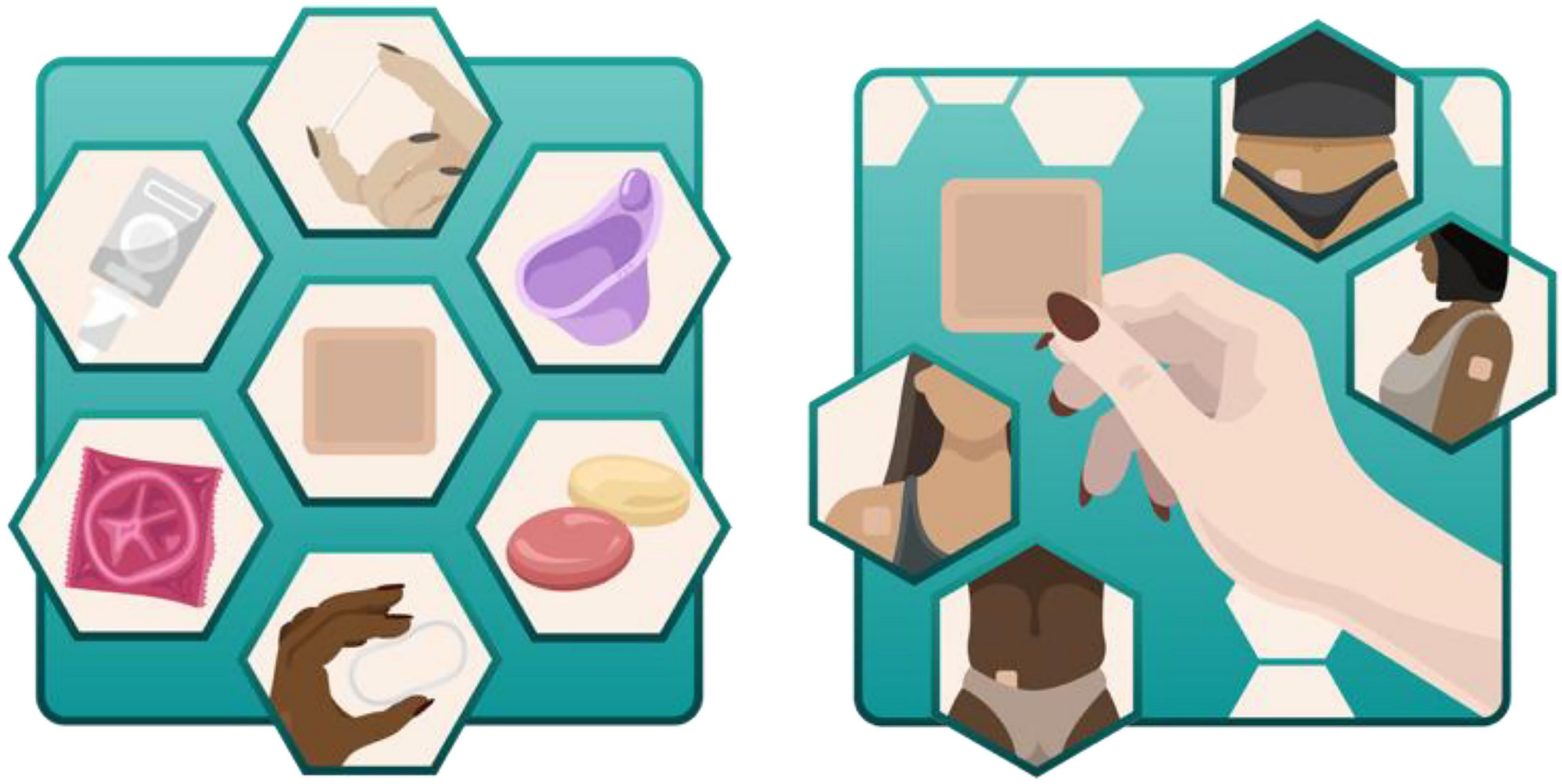
Example of inclusive designs by Catie Varley (co-author).

Visual design operates not just for aesthetic appeal but also as a form of ethical communication. The results reveal a tension: clinical aesthetics were associated with legitimacy and research credibility yet also carried negative connotations and were seen as visually unappealing, while a commercial style raised fears of data exploitation. T.H.E app, and all technology-enabled care, exists within a broader ecosystem of available and actively used technologies. How the visual design reflects these will carry connotations that may shift over time and in response to emerging external contexts. This argument brings well-established understanding of visual language and design to bear on technology-enabled data systems. Results suggest that visual design will impact use and acceptability due to appeal, relatability, and humanisation. Seeing oneself, through representation of images, values and lived experiences, can increase relatability and engagement (37), while desire for humanisation of the project and T.H.E app may present accountability and relational presence, countering the anonymity of digital systems. Within the app's redesign, particular focus has been given to this balance in design and branding and illustrations have been created that represent diversity, an example can be seen in Figure 4. These results highlight the importance of considering and embedding visual design and branding as part of trust building, essential to engagement and use within technology-enabled data systems. They also illustrate that visual design communication is a balance and provides the art of communication, not simply colour schemes for appeal. This illustrates the need for valuing the skills of designers who can use and craft visual language.

### 4.5 Embedding meaningful value for the user into data systems

Results highlighted that the participants wanted personal value from the app including presentation, visualisation, and medical interpretation of their data, as well as the incorporation of health resources. This resulted in change from initial design scope and focus on producing research data to a wider design scope that integrates data presentation, visualisation, and insights alongside reliable information about hormonal contraception to encompass providing direct benefit to the user. Inputting data requires work, time, and effort and, as illustrated in the workshops, is potentially an emotional burden for the user (33, 38). It should be understood as a form of labour. Therefore, researchers should move away from extractive practices and the historic context of devaluing the labour of women, and instead prioritise meaningful value for the user. Since data is embodied, it might be considered to belong to its subject and should therefore be accessible to them in a clear and meaningful way.

Functionalities proposed by participants provide opportunities for technology-enabled data systems to support individuals to better understand their own health and bodies, make decisions about hormonal contraception informed by their personal data and improved understandings, and equip them with records to support conversations and consultations with health care professionals (12). This contributes to ongoing discussions of the potential of digital health tools to support informed contraception decision-making and personal autonomy, by offering knowledge and enabling personal sense-making (12, 33, 39). As highlighted by participants, if this is done with cultural sensitivity and a diverse representation of experiences, it has the potential to address gaps in access to care caused by personal circumstances, family and social structures, cultural taboo, and cultural normalisation of adversity surrounding a certain topic of women's health (12).

### 4.6 Methodological implications: feminist co-design as a more equitable process and outcome

The use of a feminist co-design methodological approach enabled the inclusion of perspectives that are often marginalised in the fields of research and digital technology development. Co-design is applied as an ethical philosophy in which those who are impacted by a product, service, or system need to be heard and have power to shape them (17). Co-design challenges existing power structures (13, 17), and is shown here to challenge scientific hegemony, patriarchy, and racial prejudice within design. Cleghorn (40) argues that, within a field that has historically dismissed women's voices, ’speaking out about your own body is profoundly feminist’. This research was conducted with the intention to create spaces in which women can speak, be listened to, and have their views acted upon through design.

The presence of critical feedback and creative input suggests that the approach created conditions in which participants felt able to challenge assumptions and contribute their perspectives and ideas as knowledge holders. The use of this methodology highlighted intersectional nuances and tensions, such as culturally situated language, the importance of data security within the context of embodied data, tension between scientific data and valuing the diversity of lived experiences, and the reciprocal value of data, where research practice cannot presume it simply has the right to women's bodily data. Feminist co-design approaches that centre on valuing the voices of marginalised groups enable tensions, assumptions, and blind spots to be identified and addressed during early stages of design and development. This can result in data collection systems and wider technology-enabled care, and femtech that better reflects and responds to the needs of diverse populations, enabling the creation of inclusive datasets and addressing health inequity. These approaches also have the potential to contribute to, and drive paradigm shifts by challenging medical and gendered norms that are often embedded within, and obscured by, claims of medical objectivity.

### 4.7 Study limitations

While the study offers valuable contributions through the exploration of T.H.E app, several limitations should be acknowledged. Although the low-fidelity nature of the paper-based app prototype supported early engagement and input, this limited the ability to assess dynamic interaction, long-term use, and the resulting data output. Advancing to a higher-fidelity prototype will enable testing within everyday contexts of use, which may yield different patterns of engagement and more developed insights into how digital tools function within lived practices. Longer-term engagement will also allow examination of the relationship between menstrual cycle symptoms and hormonal contraception side effects, as well as differentiation between them. Exploring this from a user engagement, understanding, and data output perspective was not possible in the current study, and user feedback may have been conflated. There are notable similarities in symptoms, such as headaches and mood fluctuations, alongside direct effects of contraception on menstrual cycles. However, analysis of longer-term patterns and symptom changes may support understanding of how hormonal contraception influences the menstrual cycle and its symptoms, while also distinguishing this from hormonal contraception side effects, at both the individual and population level. Further exploration is needed to determine how menstrual cycle tracking should be incorporated into future design iterations of the T.H.E. app.

While collaborative settings offered benefits in supporting an iterative and collaborative building of understanding, it is acknowledged that this may have impacted participants’ comfort level to share what may feel sensitive, private, and taboo subjects in relation to hormonal contraception. It is acknowledged that paper-based prototypes do not replicate the dynamic interactions of a digital interface; this limitation was considered appropriate at this early stage of development, where openness to fundamental design change was prioritised over the fidelity of experience.

Although the sample included diverse participants, the study does not encompass the full spectrum of minority experiences, and the UK-based context reflects specific cultural, political, and regulatory conditions that may shape perceptions of reproductive health data collection. While the findings have broader implications for technology-enabled data systems, they are derived from the development of a single digital tool focused on hormonal contraception side effects, which may limit transferability to other areas of women's health. Future design and development will aim to address these limitations through iterative development and testing of the redesigned app, engagement with diverse communities, and integration into clinical pathways.

### 4.8 Implications for future research and practice

The presented findings offer implications for the design of women’s health data collection systems within technology-enabled care and femtech, which are summarised in Table 2.

**Table 2:** Summary of co-design findings implications for research and practice.

Co-design finding	What it reveals about women's health and contraception data collection	Implication for technology-enabled care and femtech
Questions about identity did not reflect diversity and intersectionality in gender and ethnicity.	Data systems can materialise and reinforce data gaps by embedding identity categories that do not reflect lived realities of identity.	Embed flexible and inclusive identity data collection not limited by pre-set and pre-identified identity models.
Data collection was not experienced as a neutral practice but as emotional and culturally situated.	Data associated with women's bodies is embodied and may be associated with grief, legality, and cultural meaning.	Adopt trauma-informed and culturally responsive data collection practises particularly when collecting related to pregnancy, pregnancy loss and termination, birth mode, mental health, and contraception use.
Participants expressed desire for information on why data is being collected.	Explanation of data purposes positions users as active contributors to knowledge who can make informed choices about the contribution of their personal and sensitive data, rather than passive data sources.	Provide easily understandable and identifiable information on what data is collected to support informed consent, meaningful engagement, and non-extractive practices.
Participants expressed concerns with data use and commercialisation.	Trust in women's health is shaped by data governance, management and use alongside surrounding political and cultural contexts surrounding data.	Ethical technology enabled care requires responsible data use, ownership, governance, and transparent communication of these to the user.
Medical terminology and question framing were often inaccessible.	Language that reflects clinical terminology, and is unclear in reasoning can create barriers to use and compromises data quality.	Ensure readability and understandability within technology-enabled care to support equitable use and valid patient generated data.
Participants wanted personal value from the app, including meaningful presentation of data opportunities for learning and medical interpretation.	Users seek reciprocal value when contributing data rather than participating in purely extractive data practices.	Move from extractive data practices to reciprocal value has the potential to support users in understanding their bodies, making informed choices and data informed clinical consultations.
Participants wanted visual design and branding to be appealing, diverse and relational while also communicating trust.	Visual design and branding impacts aesthetic appeal while also communicates values and ethical positioning therefore it impacts engagement, acceptability, and trust.	Treat visual design and branding as a mechanism for trust building, relationality and engagement with technology enabled care rather than as a secondary aesthetic consideration.

These findings emphasise the embodied, emotional, and culturally situated nature of women's health data, underscoring the need for trauma-informed and culturally responsive approaches to data collection. They highlight the limitations of clinical and medical framing that can be harmful and does not reflect the lived realities of women. The findings also present tensions that while categorisation enables analysis of the potential impacts of gender and ethnicity on hormonal contraception side effects, that introducing closed categorisation in data collection can miss diverse identities and experiences, embedding blind spots and reinforcing existing gaps. Future research should prioritise developing flexible and inclusive measurement approaches, supported by digital systems capable of capturing diverse and emergent forms of experience.

In parallel, for all uses of technology-enabled data collection systems, data security, governance, and ethical practice must be imperative and embedded throughout design. Data security and ethical use are reflected not only in digital architecture and data management, but also in data commercialisation, secondary use, and how or whether this is communicated to the user. Data collection should be communicated to users clearly and accessibly, not hidden in terms and conditions or buried in difficult-to-understand language. This enables informed consent that truly values the decisions of women. Within data collection systems, language, readability, and visual design should be treated as core components of system design, shaping not only usability but also trust, engagement, and equity.

Data collection systems should provide value to the user, to reflect and embed non-extractive practices. Participant suggestions highlight opportunities to support women in learning about and understanding their bodies and reactions to hormonal contraception, as well as in accessing help and medical intervention when needed. T.H.E application and similar femtech products could offer an additional pathway for support, extending beyond data capture alone. Future research should explore the integration of T.H.E app into clinical pathways. This could explore and optimise the design for the specific context and user experience it would sit within and examine the app's impact on data completeness and support for women's health outcomes. Together, this work could contribute to addressing persistent data gaps in hormonal contraception, informing future research, while aligning digital health technologies with more ethical, user-centred data practices.

By embedding culturally responsive, trauma-informed and ethical data design principles, T.H.E app has the potential to empower users by providing an accessible, inclusive, and engaging environment in which they can better understand, make decisions and have informed conversations about their own health. Beyond individual benefits, more equitable engagement with the app could generate more representative data on hormonal contraception side effects, addressing longstanding systematic gaps in understanding to inform future healthcare practice and research. As such, this approach has the potential to reduce health inequities at both the individual and systems levels, while providing a framework for designing more equitable technology-enabled care across other areas of women's health.

## 5 Conclusions

The study offers implications for the design of technology-enabled data collection systems to support more equitable and ethical data practices associated with women's health and hormonal contraception. This suggests that technology-enabled care can support individual health while also addressing data gaps and the inequities embedded in existing data and design.

This study has used feminist co-design methods to value, reflect, respond, and embed the voices, values, and ideas of those from minoritised communities in the design and development of T.H.E app. Participant insight translated directly into design decisions: identity questions were made flexible rather than fixed, the ONS 4 well-being measure was replaced with a less clinical, less triggering alternative, sensitive questions on abortion, pregnancy loss, and mental health were reworded and made optional, clinical terminology was replaced with plainer, warmer language throughout, and features for data visualisation, personal insight, and culturally relevant health information were added. Together, these changes illustrate how co-designed insight can be translated into concrete product decisions, offering a model for inclusive digital health design that moves femtech beyond commercially-driven, one-size-fits-all norms toward tools that are trauma-informed, culturally responsive, and reciprocal.

For the purpose of open access, the author has applied a Creative Commons Attribution (CC BY) licence to any Author Accepted Manuscript version arising.

## Data Availability

Excerpts from the original contributions are presented within the study. Full original contributions are not shared due to the nature of conversations being at times personal and sensitive. Further inquiries can be directed to the corresponding author.
